# The dose of remimazolam combined with sufentanil for the induction of general anesthesia in obese patients undergoing bariatric surgery: an up-and-down sequential allocation trial

**DOI:** 10.3389/fphar.2024.1411856

**Published:** 2024-09-25

**Authors:** Minghui Chen, Huiying Wang, Jiajun Sun, Tao Zhang, Xiaoyin Niu, Tingting Zhang, Jian Liu, Xuan Zhao

**Affiliations:** Department of Anesthesiology, Shanghai Tenth People’s Hospital, Tongji University School of Medicine, Shanghai, China

**Keywords:** remimazolam, sufentanil, induction, up-and down allocation, obesity, lean body weight

## Abstract

**Background and purpose:** Remimazolam is a newly developed benzodiazepine drug with water-soluble, esterase degradation, and ultra-short-acting properties. The dose for general anesthesia induction in obese patients was not known. This study aimed to determine the optimal dose of remimazolam in combination with sufentanil for the induction of general anesthesia in obese patients.

**Methods:** It was a prospective observational study. We recruited 46 patients scheduled for bariatric surgery from October 2022 to December 2023. One patient refused to provide informed consent, and six patients were receiving psychotropic medication. Thirty-nine patients were enrolled. The Modified Observer’s Assessment of Alertness/Sedation (MOAA/S) scale was used to assess the patient’s response. The dose of sufentanil was 0.5 µg/kg (lean body weight [LBW]). The initial dose of remimazolam was 0.3 mg/kg (LBW). The dose of remimazolam was modified using the up-and-down allocation technique. Successful sedation (negative group) was characterized by achieving a MOAA/S score ≤ 1 within 3 min of commencing remimazolam infusion. If negative, the next patient received a low-level dose at a ratio of 0.9. Failed sedation (positive group) was defined as a MOAA/S score of >1 within 3 min of commencing remimazolam infusion. The patients in the positive group received propofol 0.5 mg/kg as a remedial measure, and the next dose was increased to a higher level. The primary outcome was to determine the half-effective dose (ED_50_) and 95% effective dose (ED_95_) of remimazolam in combination with sufentanil 0.5 µg/kg for induction in obese patients. The secondary outcome was to determine the occurrence of adverse effects such as hypotension, hypertension, and intraoperative awareness.

**Results:** The ED_50_ and ED_95_ values of remimazolam (LBW) combined with sufentanil (0.5 µg/kg) (LBW) were 0.115 mg/kg (95% CI: 0.072–0.137) and 0.179 mg/kg (95% CI: 0.150–0.434), respectively, and the time of loss of consciousness in the negative group was 120.13 ± 25.03 s. The cardiovascular system was stable during the induction period. The incidence of post operative nausea and vomiting (PONV) was 38.5% in 39 patients. Respiratory depression, allergic reaction, intraoperative awareness, and delayed emergence were not observed in any patient.

**Conclusion:** Remimazolam combined with sufentanil (0.5 µg/kg) (LBW) can be effectively used for general anesthesia induction in obese patients. The ED_50_ and ED_95_ values of remimazolam (LBW) were 0.115 mg/kg and 0.179 mg/kg, respectively.

**Clinical Trial Registration**: www.chictr.org.cn, identifier ChiCTR2200065602.

## 1 Introduction

According to the definition of obesity by the World Health Organization (WHO), a body mass index (BMI) exceeding 30 kg/m^2^ is considered obese (World Health Organization, 2021). In 2016, 13% of the world’s population was categorized as obese (Seidell and Flegal, 1997). The prevalence of obesity is increasing, correlating with higher occurrences of gallbladder diseases, osteoarthritis, and certain cancers than those in non-obese individuals. Consequently, an increasing number of obese patients undergo surgical procedures and necessitate general anesthesia.

Remimazolam, a newly developed benzodiazepine drug, exhibits ultra-short-acting properties with a maximum sedative effect achieved within 3 min of administration (Doi et al., 2020). It has been used successfully for sedation and anesthesia, even in special patients, such as pediatric patients (Pieri et al., 2024) and cardiac patients undergoing non-cardiac surgery (D'Andria et al., 2024). It is a water-soluble, ester-based drug that is rapidly degraded by tissue esterases to an inactive metabolite, which may be a pharmacologically appropriate choice for obese patients. However, the induction dose of remimazolam combined with sufentanil for general anesthesia in obese patients is still elusive. The dose allocation technique, known as the up-and-down method, was frequently used to calculate the dose based on the previous patient responses (Oron et al., 2022; Pace and Stylianou, 2007). In this study, we used the up-and-down method to determine the half-effective dose (ED_50_) and 95% effective dose (ED_95_) of remimazolam for anesthesia induction in combination with sufentanil (0.5 μg/kg) in obese patients undergoing bariatric surgery. Furthermore, the hemodynamic changes and adverse effects such as nausea and vomiting and delayed emergence were observed.

## 2 Materials and methods

### 2.1 Study design and participants

The study obtained approval from the Ethics Committee of Shanghai Tenth People’s Hospital of Tongji University (Shanghai, China, SHSY-IEC-4.1/21-257/01) and was conducted from October 2022 to December 2023, registered at www.chictr.org.cn (ChiCTR2200065602). All patients provided written informed consent before participating. As per the modified Dixon’s up-and-down method, 6 crossover pairs in the same direction and at least 20 or more patients were necessary. Patient enrollment in this study continued until nine success-to-failure pairs were achieved.

Inclusion criteria comprised patients aged 18–60 years, scheduled for bariatric surgery, with a BMI of 30–40 kg/m^2^, and an American Society of Anesthesiologists status (ASA) of Ⅰ–Ⅲ. Exclusion criteria included incapacity to provide informed consent, chronic heart failure (NYHAⅡ-Ⅳ), uncontrolled hypertension (systolic blood pressure >180 mmHg or diastolic blood pressure >110 mmHg), significantly impaired hepatic or renal function (Child-Pugh B and C or estimated glomerular filtration rate <90 mL/min), difficult mask ventilation, preoperative cognitive impairment, mental disease (such as depression and anxiety), use of psychotropic drugs (antidepressants and anxiolytic drugs, including benzodiazepine), and pregnant and lactating women.

### 2.2 Procedures

All patients did not receive premedication and were routinely fasted before surgery. Routine monitoring included noninvasive blood pressure, heart rate, electrocardiogram (ECG), and pulse oximetry after entering the operating room. The lean body weight (LBW) was calculated according to the formula provided by the WHO. Internal jugular vein catheterization was performed under local anesthesia. Invasive blood pressure was monitored, and 6 mL/kg (LBW) lactated Ringer’s solution was infused 30 min before induction. Remimazolam was diluted with normal saline to a concentration of 1 mg/mL. The patient’s loss of consciousness (LOC), as evidenced by unresponsiveness to shoulder stimulation, was assessed using the Modified Observer’s Assessment of Alertness/Sedation (MOAA/S) scale. The time to LOC was the time from the beginning of the remimazolam injection to MOAA/S ≤ 1.

Patients received mask oxygen supplementation at a flow rate of 10 L/min during induction. After a 5 mg dexamethasone injection, anesthesia induction began with a bolus of 0.5 µg/kg (LBW) sufentanil administered slowly over the course of 30 s. Then, remimazolam was infused steadily for 2 min, which was determined by up-and-down allocation (see below Section 2.3). When the MOAA/S score was ≤1, a bolus of 0.6 mg/kg rocuronium bromide was injected, and tracheal intubation was performed 90 s later. The hemodynamic changes were observed for 5 min after intubation.

Anesthesia was maintained with sevoflurane, dexmedetomidine, remifentanil, and rocuronium. Thirty minutes before the end of the surgery, 1 mg droperidol and 5 µg sufentanil were given intravenously. Fifteen minutes before the end of the procedure, rocuronium was discontinued. Five minutes before the end of the procedure, sevoflurane was discontinued. At the end of the surgery, the trocar sites were infiltrated with 0.5% ropivacaine (10 mL), and remifentanil was discontinued. A bolus of 2 mg/kg sugammadex was administered to facilitate extubation, and then the patients were transferred to the post-anesthetic care unit. If nausea or vomiting occurred, 10 mg metoclopramide was injected.

The mean arterial pressure (MAP) and heart rate (HR) were recorded at the following time points: at the beginning of sufentanil injection (T_0_), 1 min after sufentanil injection (T_1_), at the beginning of remimazolam infusion (T_2_), 1 min after remimazolam infusion commencing (T_3_), 2 min after remimazolam infusion commencing (T_4_), 1 min before intubation (T_5_), at the time of intubation (T_6_), 1 min after intubation (T_7_), 2 min after intubation (T_8_), 3 min after intubation (T_9_), 4 min after intubation (T_10_), and 5 min after intubation (T_11_). Adverse reactions were recorded, including hypotension, hypertension, bradycardia, tachycardia, respiratory depression, PONV, allergic reaction, intraoperative awareness, and delayed emergence. Hypotension was defined as the MAP decreased by 30% or more than the baseline value (T_0_). Hypertension was defined as the MAP increased by 30% or more than the baseline value (T_0_). Tachycardia was defined as the HR above 20% of baseline or 100 beats/min. Absolute bradycardia was defined as the HR less than 50 beats/min. Respiratory depression was defined as SpO_2_<90%. The investigators adjusted the anesthetics, concomitant drugs, and fluid infusions to maintain a systolic blood pressure ≥ 90 mmHg and ≤ 140 mmHg and heart beat ≥50 bpm and ≤100 bpm.

### 2.3 Up-and-down allocation

The dose of remimazolam and sufentanil was determined using LBW. The initial dose of remimazolam was 0.3 mg/kg (Dai et al., 2021). The dose of remimazolam was modified using the up-and-down allocation technique based on the previous patient’s response. Successful sedation (negative group) was characterized by achieving a MOAA/S score ≤1 within 3 min of commencing remimazolam infusion. If negative, the next patient received a low-level dose at a ratio of 0.9. Failed sedation (positive group) was defined as the MOAA/S score of >1 within 3 min of commencing remimazolam infusion. The patients in the positive group received 0.5 mg/kg propofol as a remedial measure, and the dose of the next patient was increased to a higher dose.

## 3 Outcomes

The primary outcome of this study was to determine ED_50_ and ED_95_ of remimazolam in combination with 0.5 µg/kg sufentanil for induction in obese patients. The secondary outcome of this study was to observe the occurrence of adverse effects such as hypotension, hypertension, awareness, delayed emergence, and PONV in both groups.

### 3.1 Statistical analysis

Statistical analyses were conducted using SPSS 27. Data were presented as numbers and mean ± standard deviation (SD). The ED_50_ and ED_95_ values of remimazolam with a 95% confidence interval (CI) for anesthesia induction were analyzed by the probit test. Hemodynamic changes were compared utilizing a one-way analysis of variance, followed by the LSD test. A *p*-value < 0.05 was considered statistically significant. A sequential graph was generated using GraphPad Prism 8.0. The simulation of the effect–site concentration of remimazolam was conducted using Excel_PkPd Ver1.46 Lite (developed by Ryuji Nakamura).

## 4 Results

### 4.1 Patient characteristics

**FIGURE 1:**
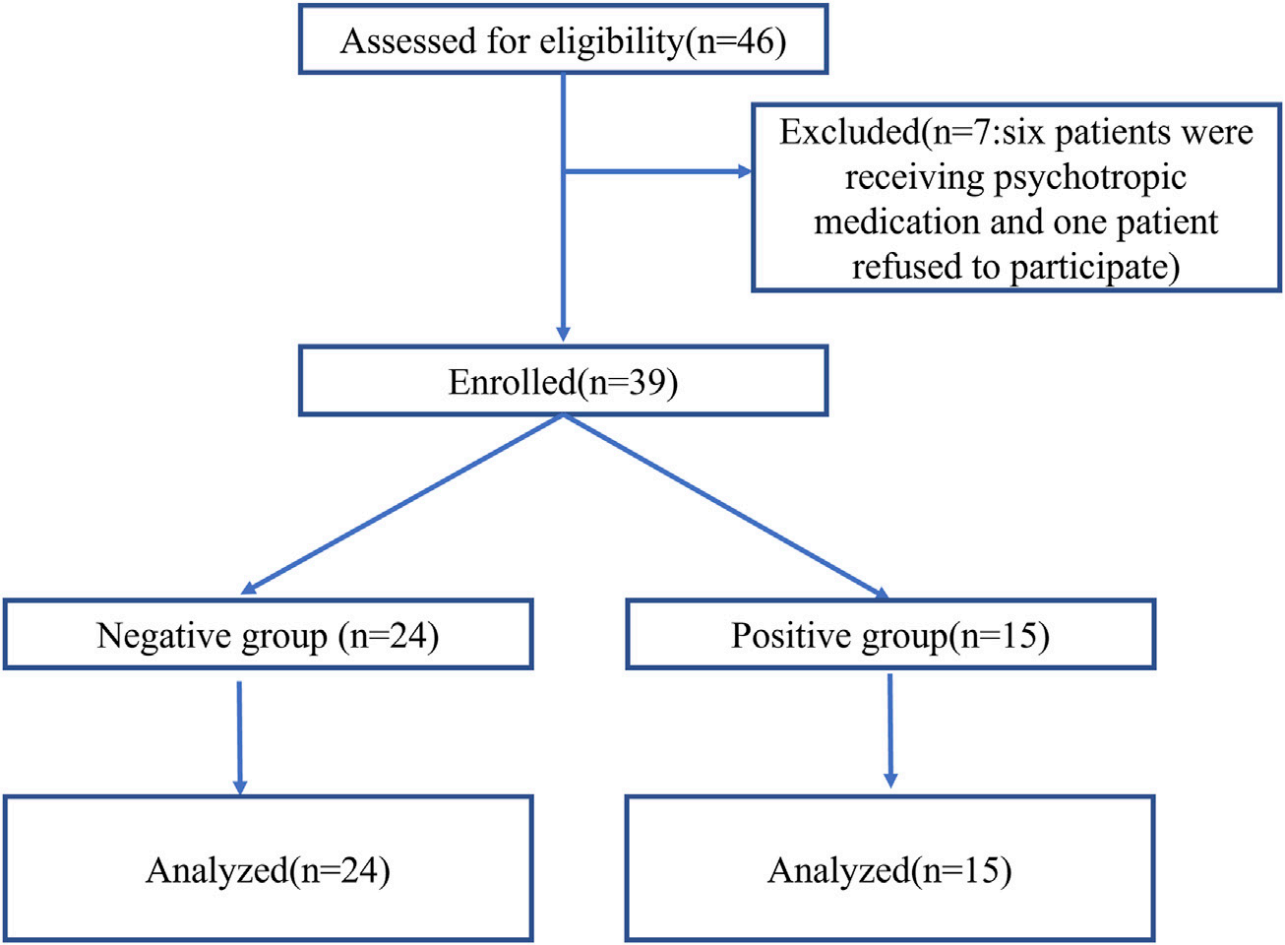
Flowchart of participants through the study.

Forty-six patients were assessed for eligibility. One patient refused to provide informed consent, and six patients were receiving psychotropic medication for depression or anxiety disorders. Thus, 39 patients were enrolled and completed the study (Figure 1). The age of patients was between 21 and 50 years. The state of “loss of consciousness” was achieved within 3 min in 24 patients (negative group) and was not achieved within 3 min in 15 patients, who then received propofol as a remedial measure (positive group). Patient characteristics are presented in Table 1.

**TABLE 1:** Demographic characteristics.

Demographic feature	All patients (N = 39)	Negative group (N = 24)	Positive group (N = 15)
Age (years)	34.33 ± 7.99	36.83 ± 7.03	30.33 ± 8.00
Male/female	8/31	6/18	2/13
Weight (kg)	97.51 ± 13.51	97.37 ± 14.28	97.72 ± 12.66
Height (cm)	166.59 ± 7.44	167.42 ± 8.78	165.27 ± 4.5
BMI (kg/m^2^)	35.04 ± 3.31	34.63 ± 3.10	35.70 ± 3.64
LBW (kg)	54.65 ± 10.13	55.49 ± 11.25	53.30 ± 8.23

BMI, body mass index; LBW, lean body weight.

### 4.2 Dose response

**FIGURE 2:**
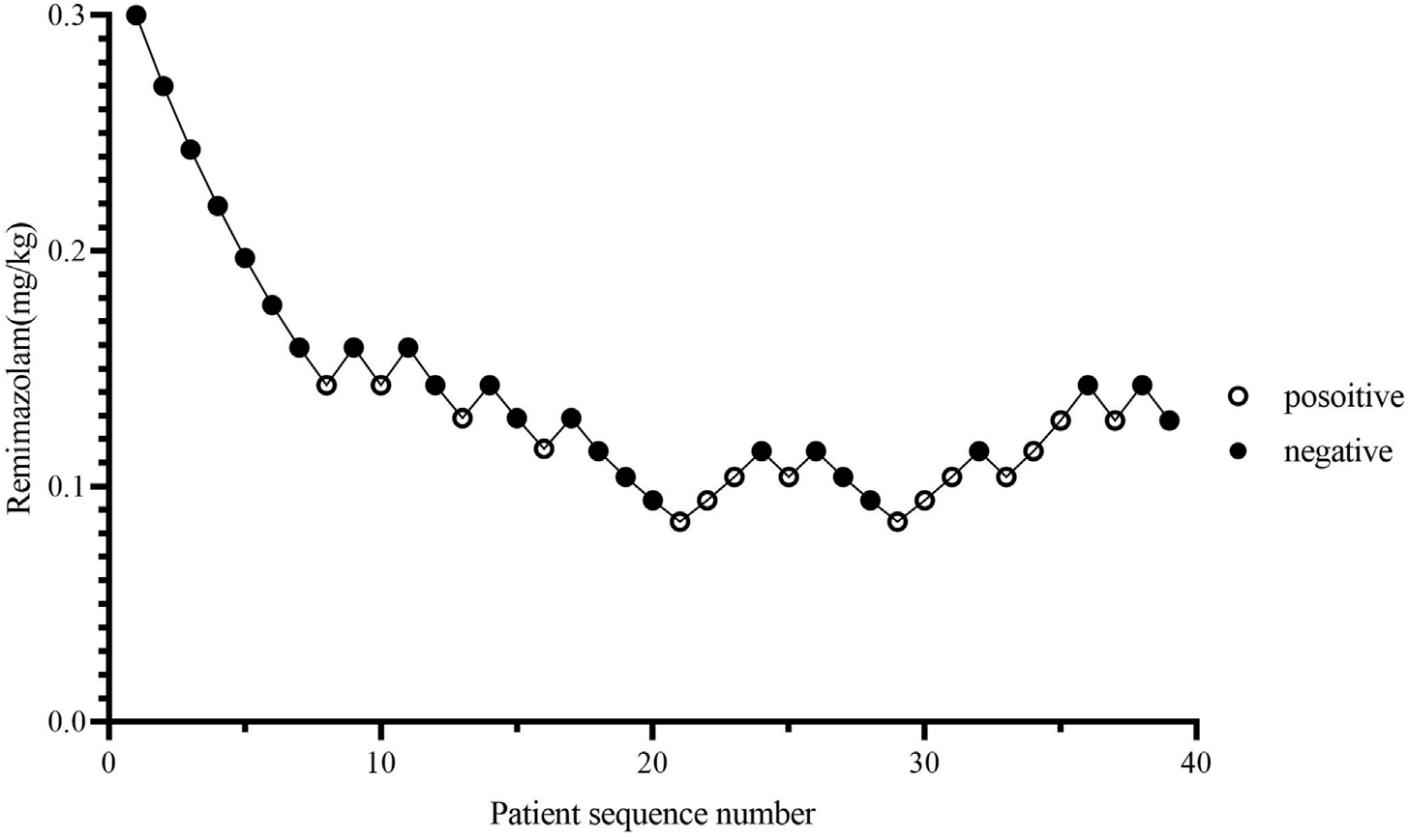
Patient response trajectory. Patient number (*x*-axis) is the exposure sequence of subjects. The dose of remimazolam (*y*-axis) was 0.3, 0.27, 0.243, 0.219, 0.197, 0.177, 0.159, 0.143, 0.129, 0.116, 0.104, 0.094, and 0.085 mg/kg. A solid circle indicates successful sedation; a hollow circle indicates a failed sedation.

**FIGURE 3:**
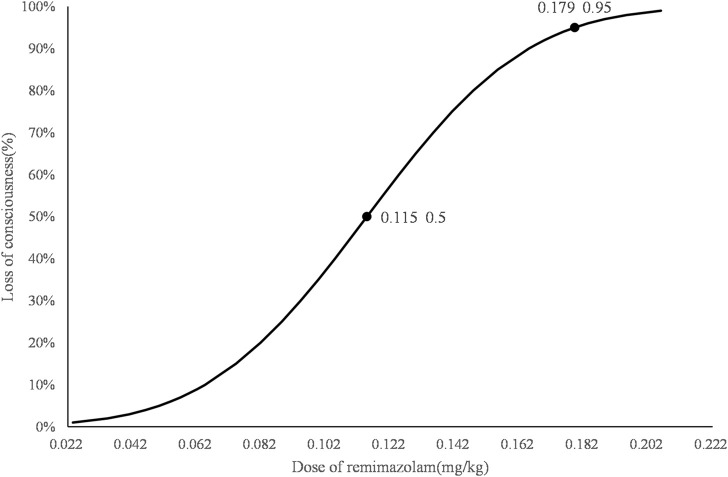
Dose–response curve for remimazolam combined with 0.5 µg/kg sufentanil for induction in obese patients. The ED_50_ and ED_95_ values of remimazolam combined with 0.5 µg/kg sufentanil to achieve the state of loss of consciousness in obese patients were 0.115 mg/kg (95% CI: 0.072–0.137) and 0.179 mg/kg (95% CI: 0.150–0.434), respectively.

The consecutive results of the infusion dose of remimazolam are shown in Figure 2. In total, nine crossover pairs appeared in this study. The ED_50_ and ED_95_ values of remimazolam combined with 0.5 ug/kg sufentanil were 0.115 mg/kg (95% CI: 0.072–0.137) and 0.179 mg/kg (95% CI: 0.150–0.434), respectively (Figure 3), and the mean time of loss of consciousness in the negative group was 120.13 ± 25.03 s. The simulated concentration of the effect site of remimazolam at the time of loss of consciousness was 283.63 ± 77.61 ng/mL in the negative group.

### 4.3 Changes in the MAP and HR in the two groups

**FIGURE 4:**
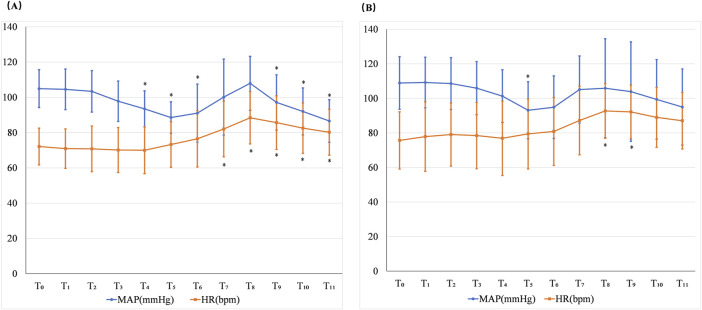
Changes in the MAP and HR in the negative group **(A)** and positive group **(B)**. T_0_: at the beginning of the sufentanil injection; T_1_: 1 min after the sufentanil injection; T_2_: at the beginning of the remimazolam infusion; T_3_: 1 min after remimazolam infusion commencing; T_4_: 2 min after remimazolam infusion commencing; T_5_: 1 min before intubation; T_6_: the time of intubation; T_7_: 1 min after intubation; T_8_: 2 min after intubation; T_9_: 3 min after intubation; T_10_: 4 min after intubation; and T_11_: 5 min after intubation. *p < 0.05 when compared with T_0._

In the negative group, the MAP statistically decreased at T_4_, T_5_, T_6_, T_9_, T_10_, and T_11_ and HR statistically increased at T_7_, T_8_, T_9_, T_10_, and T_11_ when compared with T_0_. In the positive group, the MAP statistically decreased at T_5_ and the HR was statistically increased at T_8_ and T_9_ compared with T_0_. The incidence of hypotension was 12.5%, while the incidence of hypertension was 4.2% in the negative group. The incidence of bradycardia was 4.2%, and the incidence of tachycardia was 58.3% in the negative group. The incidence of hypotension was 6.7%, while hypertension did not occur in the positive group. The incidence of bradycardia was 6.7%, and the incidence of tachycardia was 60% in the positive group (Figure 4).

### 4.4 Incidence of other adverse effects

The incidence of PONV was 38.5% (15) in 39 patients. The incidence of PONV in the positive group was 46.7% (7) in 15 patients, while the incidence in the negative group was 33.3% (8) in 24 patients. Respiratory depression, allergic reaction, and intraoperative awareness were not observed in any patient. No patient required reversal with flumazenil.

## 5 Discussion and conclusion

Owing to its pharmacological advantages (water solubility, ester structure, and rapid onset), remimazolam may be a preferred option for general anesthesia in obese patients theoretically. A small number of studies have explored the sedative safety and efficiency of remimazolam in obese patients. For sedation during gastrointestinal endoscopy, the combination of remimazolam and esketamine reduced the incidence of severe hypoxemia compared with propofol combined with esketamine (Zhang et al., 2023). For the induction and maintenance of gastrointestinal surgery, a recent study revealed that remimazolam had a lower incidence of adverse events, including bradycardia, hypotension, apnea, nausea, and vomiting, compared with the use of dexmedetomidine (Deng et al., 2024). There were also several case reports using remimazolam in obese patients (Lee and Han, 2023; Kainuma et al., 2024). In those studies, the doses of remimazolam were fixed according to the recommendation by the manufacturer; therefore, it was imperative to identify the appropriate doses for the induction and maintenance of remimazolam in obesity.

In the present study, we used the up-and-down method to determine the dose of remimazolam in combination with sufentanil for induction in obese patients. Up-and-down is the most popular dose-finding design and is used commonly in anesthesiology, which can improve the efficiency of experimental design. Allowed doses are uniformly spaced in an algebraic or geometric sequence (Liu et al., 2022). In our research, we used a geometric sequence. Considering the water-soluble property, we chose LBW as the basis of medication administration. The initial dose was 0.3 mg/kg (LBW), which was based on a previous study that showed that 0.3 mg/kg and 0.4 mg/kg remimazolam had the same success induction rate as propofol, while 0.2 mg/kg remimazolam had a lower success rate (Dai et al., 2021). After a bolus of 0.5 µg/kg (LBW) sufentanil administration, remimazolam was infused for 2 min, followed by 1-min observation. These intervals were set according to a previous study in which the mean time to LOC was 102.0 (±26.6) and 88.7 (±22.7) s when the infusion rate of remimazolam was 6 mg/kg.h or 12 mg/kg.h for 2.5 min. The dose in our study, which began at 0.3 mg/kg in 2 min, equaled the infusion rate at 9 mg/kg/h. The sedative effect of remimazolam was assessed by the MOAA/S scale in our study. LOC was defined as the unresponsiveness to shocking their shoulders, which was scaled as a score of 1 on the MOAA/S scale. The bispectral index (BIS) was also used in our preliminary study, and we found a significant correlation between the BIS and MOAA/s to monitor the sedation depth of remimazolam, which was in accordance with a recent study (Zhao et al., 2023).

In the present study, we found that the ED_50_ and ED_95_ doses of remimazolam combined with 0.5 µg/kg sufentanil were 0.115 mg/kg (95% CI: 0.072–0.137) and 0.179 mg/kg (95% CI: 0.150–0.434), respectively. Chae et al. (2022) reported that the ED_50_ and ED_95_ doses of intravenous bolus remimazolam for LOC were 0.19, 0.17, 0.14, 0.125, 0.19, 0.09, and 0.082 mg/kg and 0.33, 0.29, 0.25, 0.22, 0.19, 0.17, and 0.14 for patients of 20, 30, 40, 50, 60, 70, and 80 years older, respectively. Liu et al. (2022) demonstrated that the ED_50_ and ED_95_ doses of remimazolam were 0.088 mg/kg and 0.118 mg/kg for patients of 60–69 years older and 0.061 mg/kg and 0.090 mg/kg for 70–85 years older, respectively. Only remimazolam was used to induce LOC in both studies. Our results were lower than those reported by Chae et al. (2022), which might be attributed to the synergistic effect between sufentanil and remimazolam. Compared with the data obtained by Liu et al. (2022), the higher dose used in our study might be due to the enhanced efficacy of remimazolam in the elderly. The reason that sufentanil was given before remimazolam in our study was due to the different time-to-peak effects. It has been reported that the time-to-peak effect for sufentanil, remimazolam, and rocuronium was 3–6 min, 1–3 min, and 1–1.5 min, respectively. Thus, a bolus of 0.5 μg/kg sufentanil was given first in 30 s; then, remimazolam was infused in 2 min with 1-min observational intervals; lastly, 0.6 mg/kg rocuronium was injected; and 90 s later, tracheal intubation was performed with the ideal effect of sedation, analgesia, and muscle relaxation.

In the present study, we further simulated the concentration of the effect site of remimazolam at LOC in the negative group using Excel_PkPd Ver1.46 Lite. This program was developed by Ryuji Nakamura based on the pharmacokinetic parameters of remimazolam oriented by Kenichi Masui (Masui and Hagihira, 2022). The simulated concentration for LOC was 283.63 ± 77.61 ng/mL, which was lower than 695 ± 239 ng/mL reported by a prior investigation on the pharmacokinetics of remimazolam in healthy male volunteers (Schüttler et al., 2020). The reasons might be attributed to the combination with 0.5 µg/kg (LBW) sufentanil and the pharmacokinetic parameters being unsuitable for obese patients in our study.

In the present study, we also observed the effect of the combination of remimazolam and sufentanil on the cardiovascular system during the induction period. Considering that more than a 30% decrease in MAP could increase the risk of stroke, in the present study, more than a 30% decrease in MAP was defined as hypotension (Bijker et al., 2012). Before intubation, anesthetics often resulted in a significant depressive effect. No hypotension occurred in the negative group before intubation in the present study. The occurrence of hypotension after the induction of general anesthesia in the negative group was 12.5% in the present study, which was lower than 36.5% using the combination of propofol and sufentanil (Jor et al., 2018). In a 3-min study, the HR significantly increased compared with baseline after a bolus of remimazolam only (Oh et al., 2022). In the present study, the HR was not statistically different when compared with the baseline before intubation. Effective management of the stress response to endotracheal intubation was a key component of anesthesia induction. Qu et al. (2023) found that the ED_95_ value of remimazolam to inhibit the endotracheal intubation response in non-frail aging patients was 0.331 mg/kg when combined with 4 µg/kg fentanyl. In the present study, the incidence of hypertension in the negative group was only 4.2% and occurred at the time of tracheal intubation and 1 min after intubation. The MAP after tracheal intubation (from T_7_ to T_11_) was not statistically increased when compared with the baseline in the negative group. The HR after tracheal intubation (from T_7_ to T_11_) was statistically higher than that at the baseline but still within 120% on average in the negative group.

Therefore, 0.5 μg/kg sufentanil combined with an effective induction dose of remimazolam was effective in suppressing the stress response to tracheal intubation.

Other adverse effects such as PONV, respiratory depression, allergic reactions, and intraoperative awareness were also observed in the present study. Given the fact that remimazolam was only used for induction, the clinical value of those observations was quite limited.

In summary, the ED_50_ and ED_95_ values of remimazolam (LBW) combined with 0.5 µg/kg (LBW) sufentanil for general anesthesia induction in obese patients (BMI 30–40) were 0.115 mg/kg and 0.179 mg/kg, respectively. The cardiovascular system was stable during the induction period when an effective induction dose of remimazolam and 0.5 μg/kg sufentanil was used.

There are several limitations to our study protocol.

First, due to experimental constraints, we did not collect arterial samples for the pharmacokinetic–pharmacodynamic study of remimazolam in obesity. Second, it did not include a comparison using the total body weight in our study. Third, we did not conduct a study of the maintenance dose of remimazolam in obesity. Although no intraoperative awareness occurred in this study, it did not mean it was appropriate to use LBW to calculate the dose of remimazolam in general maintenance in obese patients. Further studies are necessary to compare the pharmacodynamic effects of remimazolam calculated from different body weights and explore the maintenance dose of remimazolam in obese individuals.

## Data Availability

The original contributions presented in the study are included in the article/Supplementary Material; further inquiries can be directed to the corresponding authors.
