# Supplementary material for: The dose of remimazolam combined with sufentanil for the induction of general anesthesia in obese patients undergoing bariatric surgery: an up-and-down sequential allocation trial
Source: Front Pharmacol. 2024 Sep 25;15:1411856. doi: 10.3389/fphar.2024.1411856 (PMC11461211; doi:10.3389/fphar.2024.1411856)
Supplement: Supplementary file 1 [file Table1.DOCX]

| **STROBE checking lists for “methods”** | |
| --- | --- |
| Study design | It’s a prospective observational study. |
| Setting | 1. The study was conducted in Shanghai Tenth People’s Hospital of Tongji University (Shanghai, China) from October 2022 to December 2023. 2. Anesthesia induction began with 0.5ug/kg (LBW) sufentanil two minutes before remimazolam. The dose of remimazolam was determined by up and down allocation and remimazolam was injected by a syringe driver for 2 minutes. The interval of begin of remimazolam and loss of consciousness was recorded. When MOAA/S score was ≤1, 0.6 mg/kg rocuronium bromide was injected and tracheal intubation was performed 90 seconds later. 3. Initial dose of remimazolam was 0.3mg/kg. Successful sedation (negative group) was characterized by achieving a MOAA/S score ≤1 within three minutes of commencing remimazolam infusion. If negative, the next patient received a low-level dose at a ratio of 0.9. Failed sedation (positive group) was defined as the MOAA/S score of ˃1 within three minutes of commencing remimazolam infusion. The patients in positive group received 0.5mg/kg propofol as a remedical measure and the dose of next patient was increased to the higher dose. 4. Record whether the patient achieved successful sedation state or not. 5. The mean arterial pressure (MAP) and heart rate (HR) were recorded at the following time points: at the begin of sufentanil injection (T_0_), 1min after sufentanil injection(T_1_), at the begin of remimazolam infusion (T_2_), 1min after remimazolam infusion(T_3_), 2min after remimazolam infusion (T_4_),1min before intubation(T_5_), at the time of intubation(T_6_),1min after intubation(T_7_),2min after intubation(T_8_), 3min after intubation(T_9_),4min after intubation(T_10_), 5min after intubation(T_11_). Adverse reactions were recorded, including hypotension, hypertension, bradycardia, tachycardia, respiratory depression, postoperative nausea and vomiting, allergic reaction, intraoperative awareness and delayed emergence. |
| participants | Thirty-nine patients aged 18 to 60 years, scheduled for bariatric surgery, with a body mass index (BMI) of 30-40 kg/m², and American Society of Anesthesiologists status (ASA) of Ⅰ-Ⅲ. |
| Variables | 1. The primary outcome of this study was to determine ED50 and ED95 of remimazolam in combination with 0.5ug/kg sufentanil for induction in obese patients. 2. The secondary outcome of this study was to determine the occurrence of adverse effects such as hypotension, hypertension, awareness, delayed emergence, postoperative nausea and vomiting. |
| Data sources/measurement | 1. MOAA/S score was used to assess the patient's sedation status. 2. MBP and HR were recorded using the monitor. |
| Bias | The patient’s sedation status was assessed by a fixed researcher. |
| Study size | As per the modified Dixon’s up and down method, six crossover pairs in the same direction and at least 20 or more patients were necessary. Patient enrollment in this study continued until nine success-to-failure pairs were achieved. |
| Quantitative variables | Quantitative datas were presented as number and mean± standard deviation (SD). |
| Statistical methods | Statistical analyses were conducted using SPSS 27. The ED50 and ED95 of remimazolam with 95% confidence interval (CI) for anesthesia induction were analyzed by the probit test. Hemodynamic changes were compared utilizing one-way analysis of variance followed by LSD test. A P value＜0.05 was considered as statistically significant. Sequential graph was generated by GraphPad Prism 8.0. The simulation of effect-site concentration of remimazolam was conducted using Excel_PkPd Ver1.46 Lite (developed by Ryuji Nakamura). |
